# Hbr1 Activates and Represses Hyphal Growth in *Candida albicans* and Regulates Fungal Morphogenesis under Embedded Conditions

**DOI:** 10.1371/journal.pone.0126919

**Published:** 2015-06-03

**Authors:** Michael L. Pendrak, David D. Roberts

**Affiliations:** Laboratory of Pathology, Center for Cancer Research, National Cancer Institute, National Institutes of Health, 10 Center Drive, Building 10, Room 2A33, Bethesda, MD, 20892–1500, United States of America; David Geffen School of Medicine at University of California Los Angeles, UNITED STATES

## Abstract

Transitions between yeast and hyphae are essential for *Candida albicans* pathogenesis. The genetic programs that regulate its hyphal development can be distinguished by embedded versus aerobic surface agar invasion. Hbr1, a regulator of white-opaque switching, is also a positive and negative regulator of hyphal invasion. During embedded growth at 24°C, an *HBR1/hbr1* strain formed constitutively filamentous colonies throughout the matrix, resembling *EFG1* null colonies, and a subset of long unbranched hyphal aggregates enclosed in a spindle-shaped capsule. Inhibition of adenylate cyclase with farnesol perturbed the filamentation of *HBR1/hbr1* cells producing cytokinesis-defective hyphae whereas farnesol treated *EFG1* null cells produced abundant opaque-like cells. Point mutations in the Hbr1 ATP-binding domain caused distinct filamentation phenotypes including uniform radial hyphae, hyphal sprouts, and massive yeast cell production. Conversely, aerobic surface colonies of the *HBR1* heterozygote on Spider and GlcNAc media lacked filamentation that could be rescued by growth under low (5%) O_2_. Consistent with these morphogenesis defects, the *HBR1* heterozygote exhibited attenuated virulence in a mouse candidemia model. These data define Hbr1 as an ATP-dependent positive and negative regulator of hyphal development that is sensitive to hypoxia.

## Introduction

Hyphal differentiation in *Candida albicans* results from an integration of signaling events from environmental cues that can act either alone or in combination. These include elevated temperature, pH or CO_2_, limiting O_2_, nitrogen or carbon, and exposure to serum, GlcNAc and other less well defined inducers. Signaling to Ras1, adenylate cyclase (Cdc35/Cyr1) and the APSES transcription factor Efg1 provides a link that integrates many of these disparate environmental signals [1–3]. Switching between hyphae and yeast maintains morphological plasticity during infection and aids commensal survival in the human host [4].

The ability to produce hyphae is essential for total virulence of the fungus [5, 6], and this process has been extensively studied [1]. Conversely, the transition from hyphae to yeast has received much less attention. This transition enables relocation to sites distal to the initial hyphal source, and provides a mechanism for vascular dispersion within the host. This may be especially important for mobilization from within a biofilm [7, 8]. *In vitro*, invasive hyphae originating from surface-grown yeast colonies have been shown to develop yeast from lateral hyphal branches. For instance, Pes1/Nop7 was recently identified using an assay to screen for mutants defective in yeast production from hyphae [7].

Hyphal-yeast conversions can also be readily observed from cells embedded in an agar matrix. However, the signaling pathways for hyphal invasion from embedded cells are distinct from those regulating surface invasion. Temperature is a major component in this process, and lower temperatures (24–30°C) favor yeast production. Farnesol, a quorum-sensing or autoregulatory factor, inhibits hyphal growth and promotes yeast conversion from pre-formed hyphae at 30°C [9].

Deletion of the known farnesol target Cdc35/Cyr1 induces hyphae formation, implicating adenylate cyclase and the cAMP-PKA signaling pathway as negative regulators of hyphae formation [10]. Indeed, both Flo8 and Efg1 are negative regulators, and both are cAMP-PKA pathway targets. Strains containing deletions of any one of these genes are filamentous under embedded conditions [10, 11].

Czf1, Mss11, and Rac1 are positive regulators of embedded hyphal growth and function in separate pathways [12, 13]. Czf1 relieves Efg1-mediated repression of filamentation, and its deletion results in the lack of hyphae under embedded conditions [12]. Mss11 functions in cooperation with Flo8 to target hyphae-specific promoters and is required for the growth of *FLO8* deletion mutants in an embedded matrix [14]. In addition, polarized hyphal growth within a matrix relies on Rho-family GTPases distinct from those used for surface expansion [3]. Rac1 and its guanine nucleotide exchange factor (GEF) Dck1 are essential for hyphae formation, indicating that the Rac1 pathway is specific for hyphal development during embedded growth [13].

Microscopically, two phenotypes of invasive hyphae are commonly encountered that result in the transition to yeast production. The first are single hyphal strands that appear to be ‘sprouting’ from deep within a colony center. A small number (<10) of these ‘sprouts’ are typically produced from a single colony, and they are distinctive due to massive accumulation of yeast that remain associated with the parental hypha [7]. In many cases the colony surface is free of other hyphae. Sprouts form rapidly (1–2 days) and are the longest of hyphal extensions and this process may aid in rapid yeast dispersion. Considering only a few sprouts are produced in a colony containing millions of genetically identical cells, this can be seen as a rare developmental event. Therefore, a novel microenvironment must drive the production of these structures. Hereafter we will refer to these structures as ‘hyphal sprouts’ due to their distinctive appearance.

The second type of hyphal structure develops as a member of a large population enclosing the founder colony. These hyphae have a uniform appearance and length and radiate from the colony center. A *flo*8/*flo*8 mutant strain or a wild type strain at 30°C embedded in YPS agar, as well as a *cpp*1/*cpp*1 strain cultured on SLAD agar illustrate this form [9, 10, 15]. Hyphal branches may or may not produce yeast and sometimes give rise to an arboreal structure in older colonies. The density of the individual hyphae can vary due according to temperature, media and incubation conditions. For instance, dense and short hyphae are produced in an *efg1/efg1* background cultured on Lee’s agar under aerobic conditions [11] or a *she1/she1* strain grown on Spider agar under a glass slide. Using the latter conditions, a wild type strain shows the other extreme: long, widely-spaced uniform hyphae [16]. Both of these hyphal types may appear together as in a Cek1 phosphatase mutant strain (*cpp1/cpp1*) grown on Spider medium at 23^°^C [17].

We have been investigating the function of an essential protein, Hbr1p, which acts as a negative regulator of white-opaque switching in *C*. *albicans*. *HBR1* heterozygosis leads to an increased rate of opaque cell formation accompanying a turnoff of the *MTL α2* gene and the ability to mate as an ‘a’ cell [18, 19]. Hbr1 possesses a nucleotide-binding P-loop that is ATP-specific and is necessary for trypsin-resistance of the purified protein [20]. The human ortholog AD-004 (CINAP, ADK6) was originally identified as a binding partner of the Cajal body component coilin. Adk6 possesses both adenylate kinase and ATPase activities [21, 22]. However, Hbr1 possesses neither of these activities [20]. The *S*. *cerevisiae* ortholog Fap7 functions in the final stages of small subunit 18S rRNA maturation. This cytoplasmic step occurs in association with an 80S-like particle containing both large and small ribosomal subunits. [23, 24].

The Hbr1 protein has a negatively charged carboxyl terminus that is the main contributor to the net negative charge of Hbr1 (calculated −47, pI 3.9) but is unique among Hbr1 orthologs. This region is predicted to be an intrinsically disordered domain that only assumes structure in the appropriate cellular context [20]. Members of this protein group include Histone H1, the Gcn4 nucleotide-binding domain, eIF4E binding proteins 1 and 2 and heat shock transcription factor [25]. Like these proteins, Hbr1 exhibits characteristic biochemical behaviors of a disordered domain protein including the lack of an informative circular dichroism spectrum, anomalous SDS-PAGE migration and a high negative charge [26–28].

We show here that a *HBR1* heterozygous mutant has decreased virulence in a mouse model and use *HBR1* mutants to assess Hbr1 function during hyphal development under embedded conditions. We show that Hbr1, like Efg1, is both a negative regulator of embedded hyphae formation and a positive regulator during aerobic invasion from the agar surface. Amino acid mutations that disrupt ATP binding lead to unique phenotypes of hyphal morphogenesis. We also identify a unique colony structure composed of unbranched true hyphae that form in the deepest layers of an agar matrix.

## Results

### Hbr1 is a negative regulator of hyphae formation under embedded conditions

Hyphal invasion through an agar matrix is an established technique in *C*. *albicans* biology to access phenotypes that may be related to tissue colonization [29]. This technique has led to the identification of a group of negative regulators of filamentation under embedded conditions. Efg1, Efh1 and Flo8 display enhanced filamentation with decreased expression [10, 11, 30]. In contrast, positive regulators such as Czf1 and Mss11 show enhanced filamentation when over-expressed, and a lack of hyphae when deleted [12, 14].

**Fig 1:**
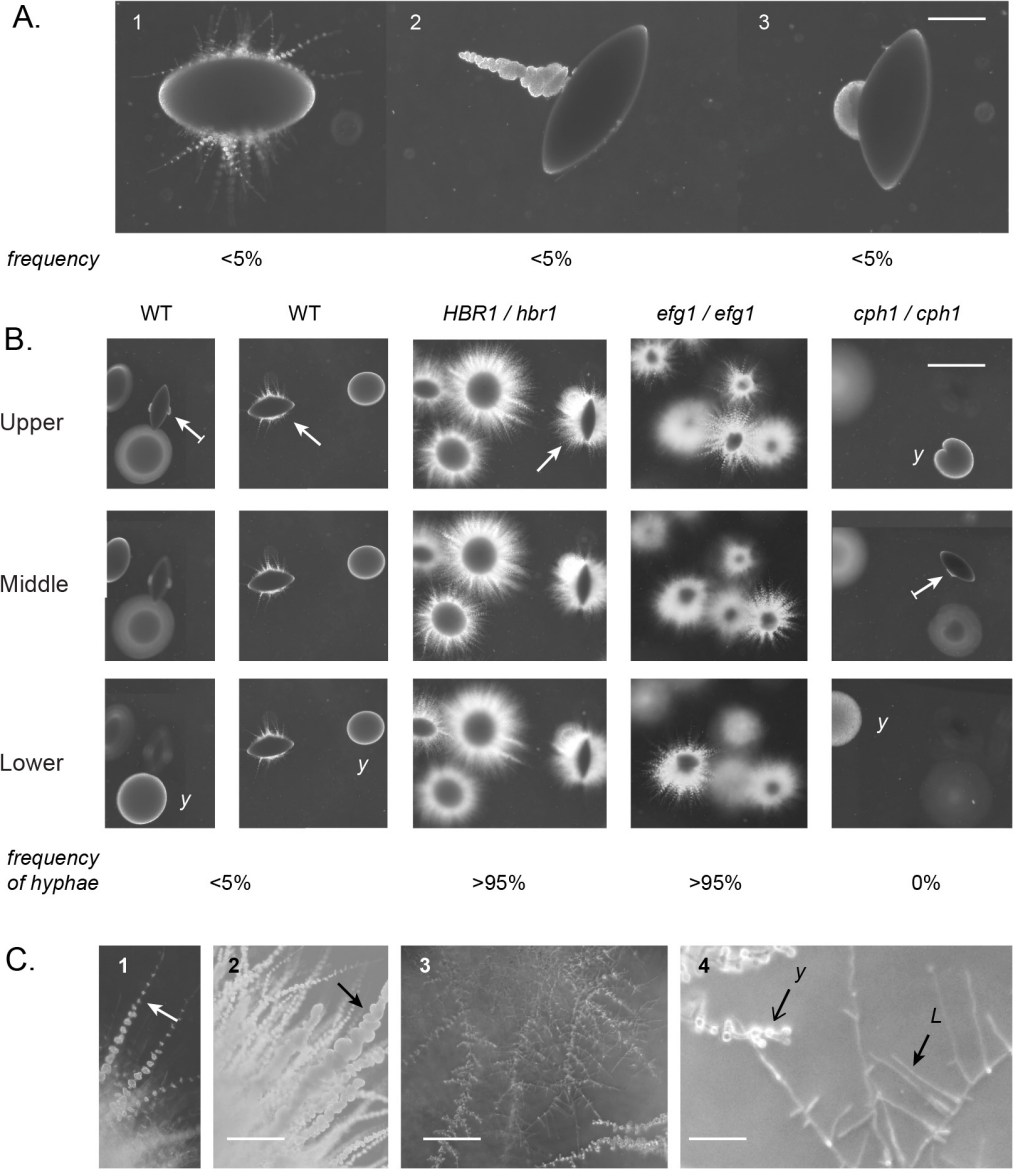
Hbr1 is a negative regulator of hyphae formation under embedded conditions. *C*. *albicans* log-phase yeast cells were embedded in molten YPS agar and incubated at 24°C. Photomicrographs were taken through the agar matrix. **(A).** Wild type strain BWP17 incubated for 4 days illustrating three modes of filamentation or yeast formation from spindle-shaped colonies. Note that these filamented colony types were only infrequently encountered. Panel 1, Pseudohyphal filaments; Panel 2, a single hyphal sprout; Panel 3, Yeast lateral outcropping from a spindle-shaped colony. Bar = 200 μm. **(B).** A comparison of colony types according to agar depth. Strains BWP17 (WT) and JKC19 (*cph1/cph1*) were incubated for 4 days; CAMPR8 (*HBR1/hbr1*) and HLC52 (*efg1/efg1*) for 2 days. The frequency that each strain formed a colony with at least 1 hypha is indicated (n >125). Strains CAMPR8 and HLC52 formed filaments at all agar depths. Surface colonies from all strains were smooth and lacked filaments. Arrow, spindle-shaped colony; arrow with line, spindle with yeast outgrowth; y, yeast colony. Bar = 2 mm. **(C).** Strain CAMPR8 cultured as above. Panel 1, simple hyphae with yeast (arrow), 2 day culture; Panel 2, hyphal sprout (arrow), 6 day culture; Bar = 200 μm; Panel 3, lateral yeast formation, Bar = 100 μm; Panel 4, perpendicular lateral branches (L) and yeast0020colonies originating at branch junctions (Y), Bar = 50 μm.

*C*. *albicans* strains heterozygous for the *HBR1* locus can differentiate to the elongated ‘opaque’ cell type, bypassing normal cellular controls that inhibit this morphogenic transition. In the processes of examining whether Hbr1 plays a role in other developmental stages, we observed an anomaly when we examined the *HBR1* heterozygous strain for hyphal development within an agar matrix. Wild type *C*. *albicans* strains embedded in agar formed two basic colony types when cultured at 24°C. In the upper levels, colonies consisted of loose aggregates of yeast that did not develop hyphae after 4 days incubation. In the lower levels, characteristic spindle-shaped colonies formed that either produced simple hyphae with few yeast, a hyphal ‘sprout’ with massive quantities of yeast, or yeast alone from a lateral site (Fig 1A, panels 1–3, resp.). In agreement with previous studies [10], hyphae formation occurred from less than 5% of the total colonies (Fig 1B, left panels). This suggested that hyphae formation in an embedded matrix is an inefficient process for wild type cells at this temperature (24°C). At 30°C more than 80% of embedded colonies have been shown to form hyphae [9].

The inefficiency of this process in wild type cells was made clear by a comparison with the *HBR1* heterozygous strain where hyphae formation from embedded colonies was greater than 95%, indicating high genetic penetrance. As early as 2 days after inoculation, *HBR1 /hbr1* cells developed hyphae at all levels within the agar (Fig 1B). These hyphae appeared with a uniform density around the initial colony and formed yeast–bearing hyphal extensions with an under layer of less well developed hyphae (Fig 1C, panel 1). When incubation was extended to 6 days, the longer hyphae became dense with yeast associated with hyphal spouts, and the under layer produced simple hyphae with yeast along their length (Fig 1C, panel 2). Incubation times of 10 days and longer resulted in arboreal structures with long, lateral branches that grew nearly perpendicular to the main filament. These hyphae were clearly distinguishable from those described above and originated away from the founder colony (Fig 1C, panels 3 and 4). Yeast developing from hyphal structures such as these have been previously described as ‘lateral yeast’, and served as an example of the conversion of hyphae to yeast production [7].

As a positive control for these experiments, we tested the *EFG1* null strain HLC52 under identical culture conditions. Interestingly, HLC52 cells formed hyphae in a pattern similar to the *HBR1* heterozygote, and filamentation was observed in >95% of the colonies at all depths within the matrix (Fig 1B). This phenocopied the *HBR1* heterozygote and occurred within a 2-day incubation period. As a negative control, deletion of Cph1 the final effector of the MAP kinase pathway for hyphae formation resulted in no filament production (Fig 1B).

The *HBR1* heterozygote and the *EFG1* null strain both produced filaments from embedded colonies at all agar depths and at high frequencies. This supports the hypothesis that Hbr1 is a negative regulator of filament formation under embedded conditions. In addition, these hyphae had a high propensity to develop yeast almost as soon as they were formed and may indicate that Hbr1also exerts control on the hyphae-yeast transition.

### Manipulation of Hbr1 results in distinctive embedded-growth phenotypes

**Fig 2:**
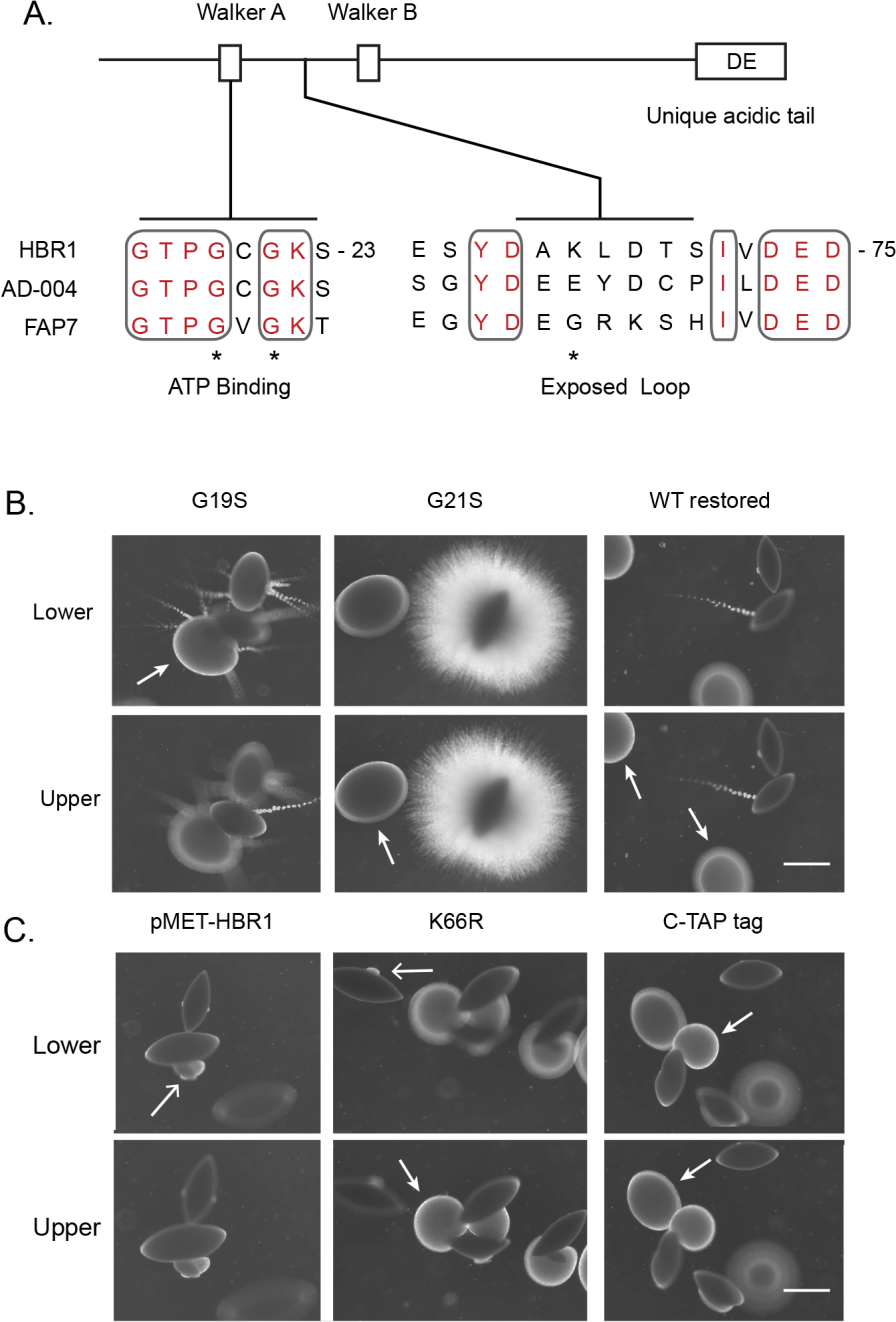
Mutations in the Hbr1 P-loop define distinct embedded phenotypes. **(A).** Locations of key predicted Hbr1 structural domains as indicated in the text. Accession numbers: Hbr1 (AF466197_1), AD-004 (NP_057367), Fap7 (NP_010115). Positions of Hbr1 mutated amino acids, (*). **(B).** Filamentation in the agar matrix can be modulated through HBR1 P-loop mutations. *C*. *albicans* cells were cultured within YPS agar at 24°C as described in the legend to Fig 1. Photographs were taken through the agar matrix at two depths. Left, G19S mutation and hyphal ‘sprout’ development from yeast colonies at all levels in the matrix except from the lower level spindle-shaped colonies. A yeast mass with developing filaments is indicated by the arrow; Middle, G21S mutation and a uniform production of simple hyphae lacking yeast from spindle-shaped colonies furthest from the agar surface; Right, strain CAMPR8 restored to wild type (Strain R8WT). Arrows indicate sites of yeast production. **(C).** Manipulation of Hbr1 by increasing copy number using strain R8MET grown without added methionine (left), mutation of solvent-exposed Lys residue using strain R866R (center) and addition of a carboxyl-terminal protein tag using strain R8CTH (right). Arrows indicate sites of yeast production. Bars = 1.5 mm.

Hbr1 binds ATP through its nucleotide binding P-loop, and mutations in this area disrupt ATP binding to the purified protein. The K22Q mutation is the most severe and is predicted to disrupt high energy phosphate contact sites, as is a G21S mutation. The G19S and K66R mutations are less severe, although disruption of ATP binding is still significant (Fig 2A) [20]. We constructed strains heterozygous for all these mutations excepting K22Q. SAT Flipper technology was used to integrate the *HBR1* constructs into the native locus, and these strains were tested for their ability to form hyphae during embedded growth as described above. Surprisingly, the P-loop mutant strains generated two different phenotypes.

The G19S mutant strain formed hyphal sprouts at all levels in the matrix with an absence of an under-layer of unbranched hyphae as seen in the *HBR1* heterozygous strain (Fig 2B). These sprouts were the source of large quantities of yeast forming away from the main body of the colony. This implies a direct dispersion effect and indicates a role for Hbr1 in the hyphae-yeast transition. Additionally, the phenotype occurred at all levels in the agar, indicating dominance of this mutation and insensitivity of this strain to hypoxia, CO_2,_ or other conditions that vary with depth in the agar. Similar to the *HBR1/hbr1* strain, the G19S mutant had high penetrance and expressivity since > 95% of the colonies displayed the identical phenotype. The G21S mutant was also dominant but showed two characteristics strikingly different from the G19S mutation. First, this strain was uniformly filamentous with a lack of hyphal sprouts, and appeared similar to the *flo8/flo8* strain described previously [10]. Second, this strain was filamentous only from spindle-shaped colonies and only in the lower third of the agar matrix (Fig 2B). This strain also showed high penetrance and expressivity since >95% of the hyphae-bearing colonies occurred at approximately the same level with the agar column. As a control for the correct integration and functioning of the SAT flipper, a non-mutated construct was integrated back into the HBR1 heterozygote to restore two wild type alleles, and the wild type phenotype was restored (Fig 2B). These data indicate that the ATP-binding activity of Hbr1 plays an essential role in filament formation and yeast dispersion, and these two phenotypes can be genetically separated. Interestingly, the *HBR1* heterozygous strain displayed both of these phenotypes simultaneously (Fig 1C, panel 1).

*HBR1* is haploinsufficient for white-opaque switching phenotype as well as for synthesis of a high affinity fibronectin receptor that is exposed by low-dose caspofungin treatment [18, 31]. This implies that Hbr1 levels within the cell are tightly controlled. When we over-expressed *HBR1* from the MET3 promoter, all embedded colonies lacked filaments. However, in the lower half of the matrix spindle colonies produced yeast from distinct lateral regions (Fig 2C, left panel). Interestingly, introduction of a K66R mutation or addition of a carboxyl-terminal TAP tag to Hbr1 produced a similar phenotype except for dense yeast foci produced throughout the matrix (Fig 2C, center and right panels). These results suggest that the K66R mutation and the C-terminal tag may lock Hbr1 into an active configuration. This may serve to increase the net Hbr1 activity, which may be also achieved through over-expression. The large scale yeast production that appears as an invasive yeast colony is most likely the result of hyphal penetration and rapid conversion to yeast.

Taken together, these results indicate that Hbr1 plays an active role in suppressing filamentation under embedded conditions at 24°C. The Hbr1 mutants define three morphological transitions: (i) dispersion through the production of a small number of hyphal sprouts that produce copious amounts of yeast, (ii) formation of massive yeast colonies that are likely the result of filaments progressing from the founder colony that produce yeast, and (iii) filament outgrowth *per se* without yeast conversion that originates from a uniform distribution of growth points from the founder colony. The *HBR1* heterozygote manifests all three colony types (Fig 1C).

### Hbr1 is a regulator of fungal tissue formation in a spindle colony

**Fig 3:**
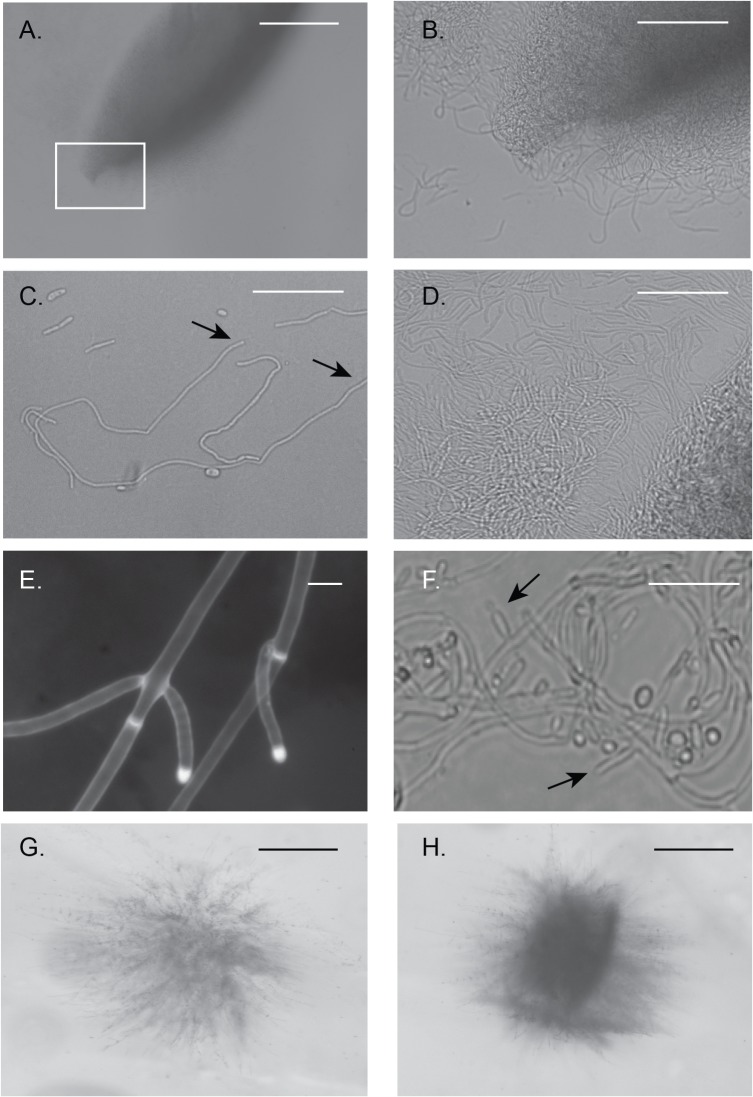
Spindle-shaped colonies reveal fungal tissue formation from embedded *HBR1/hbr1* cells. Hyphal matrix created within a spindle-shaped colony. **(A-D).** Photomicrographs through a gel slice dissected from a 4 day old embedded culture of CAMPR8 (*HBR1/hbr1*) and crushed onto a glass slide. **(A).** A spindle-shaped colony obtained from the deepest part of the agar. Bar = 500 μm (**B).** Magnification of area boxed in (A) illustrating intertwined hyphae that comprise the spindle colony. Bar = 100 μm **(C).** Isolated filaments dissected from the gel demonstrating the absence of hyphal branching. The approximate length of the longest filament measured from arrow to arrow is 350μm. Bar = 50 μm. **(D)**. An interwoven mesh of unbranched hyphae taken from a spindle colony. Bar = 50 μm. **(E).** A filament in the initial stages of branching dissected from a spindle colony and stained with calcofluor white. Note the absence of constrictions at the branch junctions and in the parental filaments, arrows. Bar = 5 μm. **(F).** Formation of hyphal branches and yeast production from a 10-day old culture. Arrows indicate selected lateral branch points. (*), yeast cell from lateral branch. Bar = 25 μm. **(G and H).** Initial stages of spindle-shaped colony formation photographed through an agar gel slice. Bar = 500 μm.

The distinctive appearance of the spindle-shape colonies described above led us to examine them in more detail by examining cross sections through agar slices. The interiors of spindle colonies from both wild type and *HBR1/hbr1* CAMPR8 cells were predominately yeast and appeared to be embedded in a matrix lacking hyphae. However, upon closer examination, a subset of the CAMPR8 spindle-shaped colonies possessed interwoven aggregates of unbranched hyphae (Fig 3A). When viewed at higher magnification, crushed colonies taken from an agar plate appeared as if the poles of the structure are formed by hyphae that have looped back into the main body (Fig 3B). At this particular stage, these hyphal elements were entirely unbranched (Fig 3C) and tightly intertwined (Fig 3D). Individual hyphae reached lengths greater than 500 μm, but we could not accurately determine lengths within a coiled hyphal mass. Fig 3C shows a representative that is approximately 350 μm in length (between the arrows). We also identified hyphae that were at the initial stages of branching and possessed true hyphae characteristics: a uniform width with parallel, uniform sides and non-constricted septa (Fig 3E).

Continued incubation up to 7 days resulted in the apparent onset of conversion of these hyphae to typical yeast cells. Initially, lateral hyphal branches formed that were competent for yeast production (Fig 3F, arrows). In later stages, more yeast cells were produced as the hyphae generated more lateral branches. We did not identify hyphae with constrictions that indicated pseudohyphae, although it is possible they exist within this dense hyphal network. We identified 10 of these colony types from the more than 60 spindle-shaped colonies we examined. Most spindle-shaped colonies were composed of densely-packed yeast cells that appeared to be embedded in a matrix material. We could not identify the direct precursors for these particular colony types but suspect that they are the result of mass conversion to yeast production within an enclosed hyphal aggregate.

Agar gel slices from strain CAMPR8 (*HBR1/hbr1*) embedded colonies also contained hyphal aggregates that appeared to identify the initial stages of spindle formation (Fig 3G). Hyphae originating from the initial inoculum spread into the matrix and then appeared to coalesce into a loose bundle of fibers. The hyphae in these bundles gathered along distinct planes suggesting some sort of organizing center or chemical gradient (Fig 3H). This process appears to be the source of the spindle-shaped colonies. We did not observe these structures in wild type colonies but we cannot exclude their production under other growth conditions.

These data indicate that the ability to form spindle-shaped colonies under embedded conditions is a distinct developmental phenotype of *C*. *albicans* that is regulated by Hbr1. The spindle colonies containing unbranched hyphae are likely a precursor to yeast-producing lateral branches. However, it is also possible that the spindle colonies could remain refractory to yeast production and exist as a distinct fungal structure.

### Distinct morphological responses to farnesol between CAMPR8 and HLC52

Farnesol is a quorum-sensing or auto-regulatory molecule produced by *C*. *albicans* in response to high cell density and other factors. It is a potent repressor of hyphal growth and exerts its effects through the direct inhibition of adenylate cyclase (Cyr1) [32–34]. Farnesol addition to wild-type strains completely abrogates filament formation *via* inhibition of Cyr1-PKA, and one target of this pathway is Efg1. In cooperation with Czf1, signaling to Efg1 is necessary to de-repress filamentation under embedded conditions, but whether farnesol suppresses the *efg1/efg1* hyper-filamentation phenotype has not been shown [9, 11]. Efg1 and Hbr1 are both regulators of white-opaque switching, and in both cases this phenotype was identified by decreasing gene dosages. Deletion of both *EFG1* alleles does not result in opaque cell production, and this procedure is lethal for *HBR1* [18, 35]. Due to the similarity of morphological responses between strains HLC52 and CAMPR8, we used farnesol to identify whether cAMP signaling similarly affects their hyper-filamentation phenotypes.

**Fig 4:**
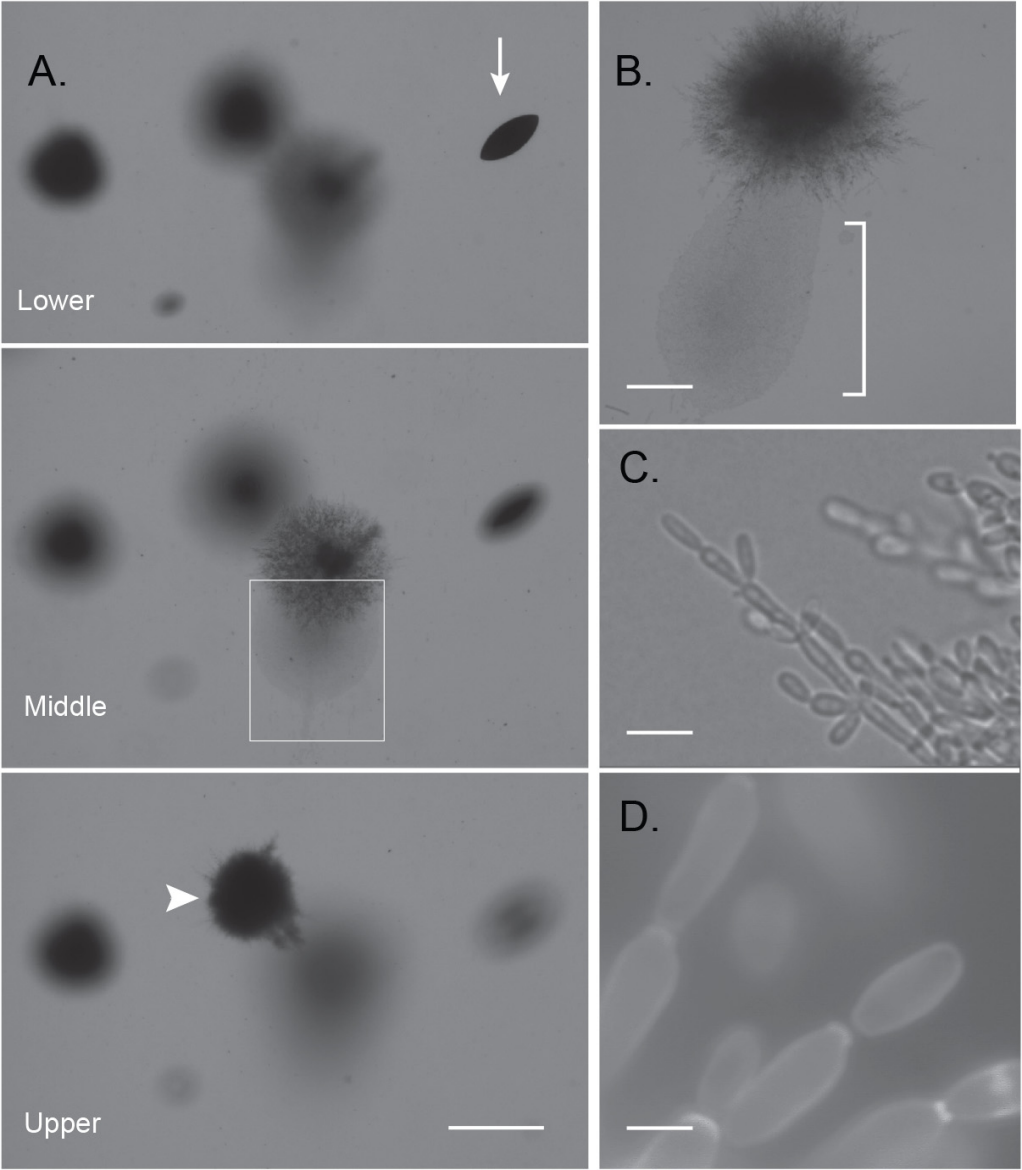
Effects of farnesol on embedded growth of an *efg1/efg1* strain. Photomicrographs taken *in situ* of HLC52 cells embedded in YPS agar containing 100 M farnesol after 2 days at 24^°^C. **(A).** Three focal planes illustrating three colony morphologies. Arrow, non-filamentous spindle-shaped colonies in the lower level of the matrix; Arrowhead, upper level colony with compact pseudohyphal filaments that do not produce yeast; Boxed region, a colony expressing arboreal-shaped branching hyphae that develop midlevel in the matrix. Bar = 2 mm. **(B).** Higher magnification of area such as boxed in (A). Masses of elongated opaque or opaque-like cells (brackets) produced through invasive pseudohyphal migration. Bar = 150 μm. **(C).** Higher magnification of a leading edge of the pseudohyphal expansion from (B). Bar = 10 μm. **(D).** An opaque-like cell aggregation illustrating the final maturation step of opaque-like cells from the pseudohyphae. Calcofluor white stain. Bar = 5 μm.

For these experiments, yeast cells were embedded into agar containing 100 μM farnesol and incubated at 24°C. Farnesol inhibited hyphae formation in control plates containing 10% serum in YP medium with 5 mM glucose, confirming its biological activity. Farnesol suppressed filamentation and enhanced yeast production in *EFG1* null cells, but the effects were depth-dependent. The spindle-shaped colonies deepest in the agar completely lacked filaments (Fig 4A, arrow). Upper level colonies were composed of short, stocky pseudohyphal filaments with elongated cells suggesting an opaque or opaque-like cell (Fig 4A, arrowhead and Fig 4D). Approximately at mid-depth, arboreal-like structures formed (Fig 4A, boxed region) that emanated massive quantities of yeast (Fig 4B, bracket). Upon closer examination, these yeast cells were opaque or opaque-like that formed from pseudohyphal-type branches (Fig 4C). Wild type cells cultured under these conditions display normal yeast that form in bunches on lateral hyphal branches [9]. Thus, the majority of filaments were clearly pseudohyphae, although at the leading edges they also appeared to have some traits of true hyphae. Elongated pseudohyphal cells could be readily found as single cell units that had separated from the main pseudohyphae and had the appearance of opaque cells similar to those previously described [35] (Fig 4D).

**Fig 5:**
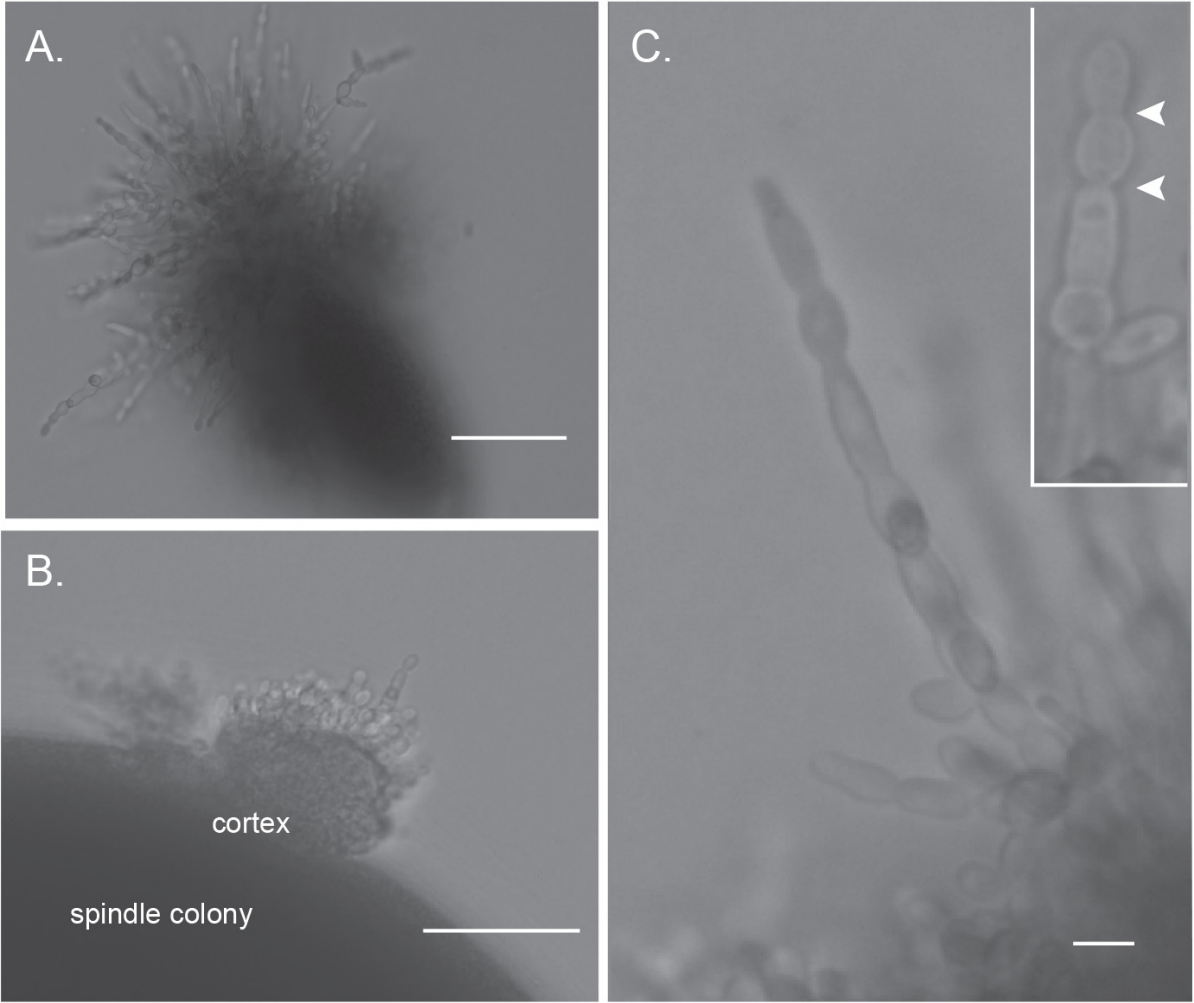
Farnesol disrupts cytokinesis and interferes with filamentous development of *HBR1* heterozygous cells embedded in agar. Photomicrographs taken *in situ* of *HBR1/hbr1* cells embedded in YPS agar containing 50 μM farnesol after 2 days at 24°C. **(A).** Filament formation from a spindle-shaped colony growing in the deepest level of the agar matrix. Bar = 500 μm **(B).** Increased magnification of (A) illustrating irregular pseudohyphal-like filaments erupting from the interior of a spindle colony. Bar = 100 μm **(C).** Higher magnifications of individual filaments illustrating defective cytokinesis. Inset, a filament illustrating infrequent septum-like constrictions (arrowheads). Bar = 5 μm.

The *HBR1* heterozygous cells treated with farnesol displayed entirely different phenotypes. In the upper layers of the agar, >90% of the colonies extended branched, irregular pseudohyphae. In the lower agar depths, filaments originating from spindle cells that had similar pseudohyphal characteristics that failed to form proper septa with constrictions (Fig 5A). The whole spindle structure appeared to enclose its contents in a compact structure composed of cells embedded in an extracellular matrix. Regions of the spindle colony sometimes erupted from the enclosure with the abnormal filaments extruded (Fig 5B).The filaments were extended but swollen and were comprised of attached cells that could not be disrupted mechanically (Fig 5C). Rounded cells alternated with extended cells suggesting defects in isotropic and polarized growth coupled with a failure in cell separation. In a minority of emanating filaments, we could see evidence of constrictions between cells, suggesting a pseudohyphal form (Fig 5C inset, arrowheads). These abnormal filaments appeared similar to *SWI1* null cells depleted of Gin4 [36].

### Hbr1 is a positive regulator of invasive hyphae production under aerobic conditions

**Fig 6:**
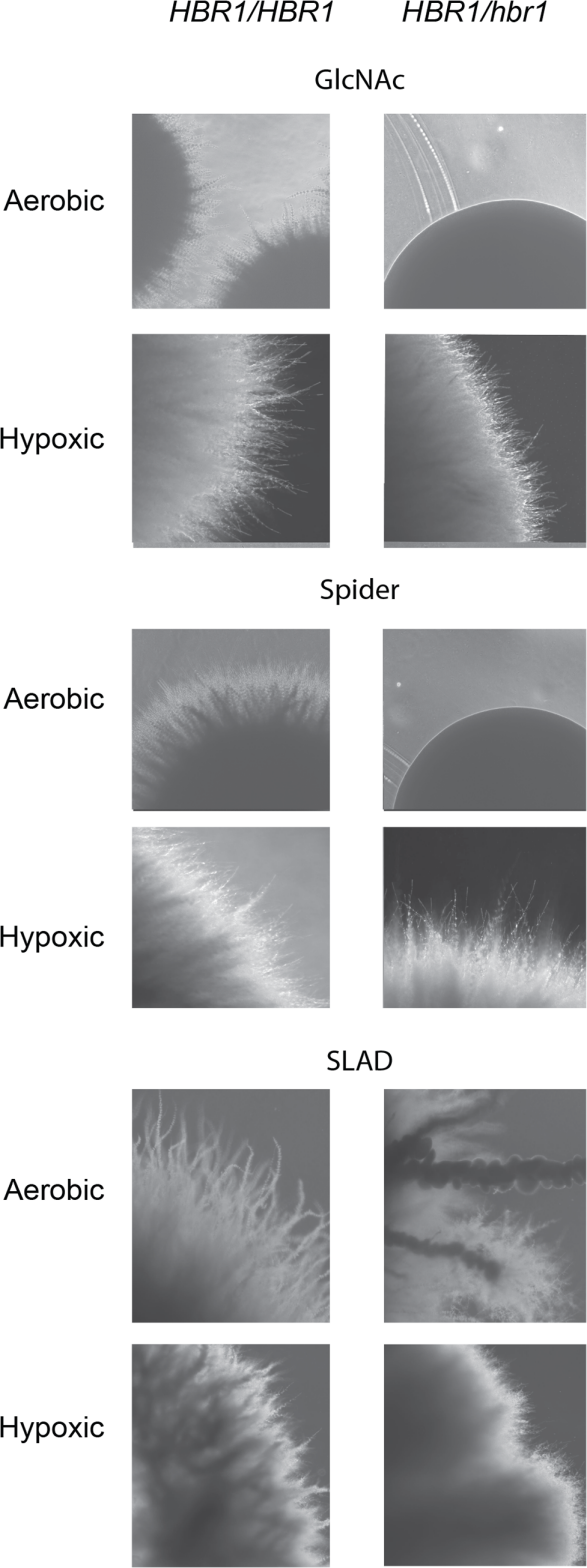
Hypoxia can rescue the agar invasion defect of the *HBR1* heterozygous strain. *C*. *albicans* strains BWP17 (left column) and CAMPR8 (right column) were cultured for 4 days on the surface of the indicated media under aerobic or hypoxic (5% O_2_) conditions at 30°C. Colonies were washed from the agar surfaces before photography through the agar.

We next assessed invasion from the agar surface under aerobic and hypoxic conditions. Interestingly, the *HBR1/hbr1* strain produced a smooth surface colony phenotype lacking invasive filaments on Spider and GlcNAc media under aerobic conditions (Fig 6, upper and center). Nitrogen limitation using SLAD agar produced a filamentous phenotype distinct from the wild type strain; instead of uniform, yeast-bearing simple hyphae, the mutant strain produced hyphal extensions dense with yeast. Interestingly, yeast attached to the main filaments also began to produce hyphae (Fig 6, lower). These data indicate that Hbr1 is required for agar surface invasion under aerobic conditions.

To reproduce the hypoxic conditions that may occur in the deeper levels of an agar matrix using these media formulations, we incubated duplicate plates at 24°C in an atmosphere of 5% O_2_, 10% CO_2_, and 85% N_2_. Surprisingly, on GlcNAc and Spider media the wild type and *HBR1* heterozygote colonies appeared identical. These colonies contained uniform distributions of simple hyphae lacking yeast (Fig 6, upper and center). Growth under nitrogen deprivation (SLAD media) showed more invasive hyphal sprouts although overall, the wild type and heterozygote colonies appeared similar (Fig 6, lower). These data indicate that Hbr1 is a positive regulator of hyphal growth under aerobic conditions but not under hypoxic conditions. This suggests that a hypoxic response acts as an *HBR1* suppressor on Spider and GlcNAc media.

The biomass beneath a yeast colony can produce carbon dioxide at levels sufficient to induce hyphae formation *via* the cAMP-PKA pathway [29, 37]. If the *HBR1/hbr1* strain was hyper-sensitive to CO_2_, this may provide a partial explanation for its filamentation phenotype during embedded growth. To test this, *C*. *albicans* yeast cells were inoculated onto DMEM agar buffered with 150 mM HEPES, pH 7 and incubated at 37^°^C in a 5% CO_2_ in an environment with atmospheric O_2_ for 72 h. These test conditions have been previously shown to induce filament formation in wild type but not adenylate cyclase null cells [37]. As expected, wild type cells formed filaments that were similar to those previously described [37]. However, the *HBR1/hbr1* strain showed a complete absence of filaments and agar invasion. The colony appearance was smooth, similar to the phenotype of this strain grown under aerobic conditions on Spider and GlcNAc media (see Fig 6 for an example of this smooth colony phenotype). This implies that CO_2_ availability is not a limiting factor for the hyper-filamentation phenotype of the *HBR1* heterozygote. Rather, this strain’s ability to form hyphae at all levels within an embedded matrix is more likely the result of O_2_ limitation.

### Hbr1 is necessary for complete virulence in a mouse model of disseminated infection

**Fig 7:**
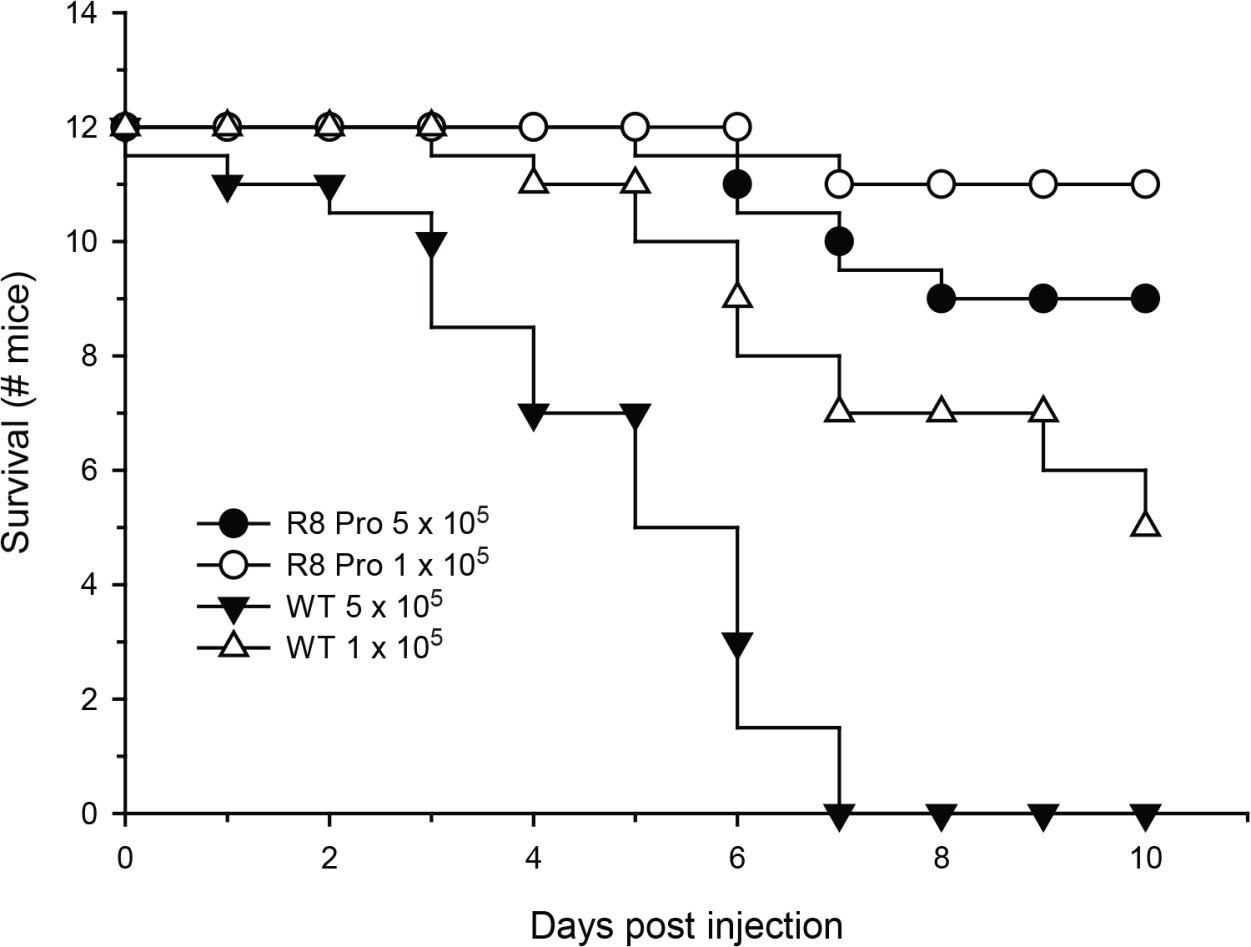
Attenuated virulence of an *HBR1* heterozygote in a mouse model of disseminated infection. Strains DAY185 (*HBR1/HBR1*) and R8PRO (*HBR1/hbr1* ARG+ URA+ HIS+ prototroph) were introduced into female Balb/c mice *via* the tail vein at the indicated doses (n = 12, each).

The ability of *C*. *albicans* to exploit diverse niches during infection is directly related to its morphological plasticity. We therefore examined whether the morphological defects seen in the *HBR1* heterozygote affect its ability to cause disseminated disease *in vivo*. We used the tail-vein model with Balb/c female mice following guidelines established for virulence testing in *C*. *albicans* [38]. The wild type strain exhibited 50% mortality after 4.5 days and is typical for this mouse strain with a dose of 5×10^5^ CFU and 9 days for 1×10^5^ CFU [38]. Mice were completely refractory to the *C*. *albicans HBR1* heterozygote challenge until day 6 and mortality never reached more than 25% (Fig 7). Therefore, a decrease in *HBR1* gene dosage by allelic deletion results in greatly attenuated virulence and indicates that Hbr1 is necessary for mouse hematogenous infection.

## Discussion

Hyphal development is a necessary component of *C*. *albicans* infections and contributes to professional phagocyte escape, tissue invasion, and biofilm development [39]. We have identified Hbr1 as a regulator of filamentation that represses hyphal development under embedded conditions but promotes agar invasion under aerobic conditions. This duality of positive and negative regulation is shared with Efg1 and Flo8, which controls a subset of Efg1-regulated genes [10, 11]. However, we have no precedent for interactions between these regulators and Hbr1. The filaments formed under embedded conditions in the *EFG1* null strain were similar to those formed in the *HBR1* heterozygote and were independent of agar depth, exhibiting parallel sides with unconstructed septa and branching pseudohyphae (Table 1). The *HBR1* and *EFG1* mutant strains displayed similar phenotypes when embedded as well as the lack of surface hyphae under aerobic conditions. These results suggest that Hbr1 and Efg1 function either in the same or parallel pathways.

**Table 1:** Colony phenotypes under various growth conditions.

Strain Notes		Genotype	Filament	Radial	Sprout	Yeast	Depth
**Embedded /24°C**							
BWP17	*HBR1/+*	+	+	+	+	all	<5†
CAMPR8	*HBR1/Δ*	+	+	+	-	all	>95
R819S	*HBR1/G19S*	+	-	+	-	midlevel	>95
R821S	*HBR1/G21S*	+	+	-	-	all	<5
R866R	*HBR1/K66R*	-	-	-	+	all	<5
R8MET	*pMET3-HBR1*	-	-	-	+	all	<5
R8CTH	*HBR1/CTAP*	-	-	-	+	all	<5
HLC52	*efg1/efg1*	+	+	-	-	all	>95
JKC19	*cph1/cph1*	-	-	-	+	all	0
**Embedded /24°C/ farnesol**							
CAMPR8	*HBR1/Δ*	+	-	-	-	all	defective ±
HLC52	*efg1/efg1*	+	-	-	-	upper	opaque
HLC52	*efg1/efg1*	+	-	-	-	midlevel	opaque
HLC52	*efg1/efg1*	-	-	-	-	lower	spindle
**Spider or GlcNAc/5%O**_**2**_							
BWP17	*HBR1/+*	+	-	-	-	surface	invasive
CAMPR8	*HBR1/Δ*	+	-	-	-	surface	invasive
**Spider or GlcNAc/atmospheric O**_**2**_							
BWP17	*HBR1/+*	-	-	-	-	surface	invasive
CAMPR8	*HBR1/Δ*	-	-	-	-	surface	smooth

(†) Per cent filamented colonies;

(±) Cell separation / cytokinesis defects

Haploinsufficiency seen with the *HBR1* heterozygous strain could be restored by complementation with wild type Hbr1 but not with constructs expressing Hbr1mutants that alter its ATP binding affinity [20]. The phenotype of the G21S mutant is remarkably similar to that of a *flo8* deletion strain cultured under similar conditions. The *flo8* deletion strain and the G21S strains form homogeneous hyphal filaments lacking yeast formation [10]. However, Cao *et al* did not indicate the depth of colony growth within the matrix. The presence of uniform radial filaments at a specific agar depth suggests that Hbr1 is regulated by specific environmental variable such as hypoxia. The G19S mutant strain formed hyphal sprouts from colonies at all depths within the agar matrix. This suggests that Hbr1 has a role in rapid and directed yeast dispersion since these sprouts produced massive amounts of yeast within 2 days. Like the *HBR1/hbr1* strain, this response was observed in the G19S mutation at all agar depths, suggesting insensitivity to O_2_ or CO_2_ concentrations. The phenotypes displayed by the G19S and G21S mutants and the *HBR1/hbr1* strains suggest that ATP binding is necessary for the function of Hbr1 bn in hyphal differentiation.

ATP binding to Hbr1 confers trypsin resistance to the purified protein indicating a conformational change [20]. It was therefore interesting that the addition of a carboxyl-terminal TAP tag resulted in a complete lack of filament production. Instead, yeast-producing colonies dominated and were similar to those of the *CPH1* null strain cultured under identical conditions. Attachment of this stable folded polypeptide tag may have altered the ability of Hbr1to adopt its natural conformation or to undergo a conformation change in response to ATP binding. Together, these results support a hypothesis that the ATP-binding P-loop of Hbr1 plays a functional role in the development of hyphae under embedded conditions at 25°C. Interestingly, the K66R charge-neutral mutation occurs in a predicted solvent-exposed loop, suggesting the alteration of a post-translational modification. Future studies will examine the post-translational regulation of Hbr1 function.

Like Hbr1, Efg1 and Flo8 are negative regulators of filamentation under embedded conditions. Efg1can be found on the *FLO8* promoter, and Flo8 and Efg1 physically interact *in vivo* and function directly on hypha-specific gene promoters [10, 40, 41]. Hbr1 possesses no predicted RNA- or DNA-binding domains [20], and so probably regulates filamentation by a different mechanism. Both Flo8 and Efg1 operate downstream of the cAMP/PKA pathway and regulate genes necessary for hyphae formation and virulence. Farnesol inhibits adenylate cyclase and wild type filamentation under embedded conditions at 30°C [9], and farnesol treatment under embedded conditions results in unique filamentation defects for the *HBR1* heterozygous strain.

Farnesol addition to the *HBR1* heterozygote under embedded conditions resulted in a consistent failure in cytokinesis. The filaments lacked a uniform thickness, lacked constrictions at what should be a bud neck, and superficially resembled pseudohyphae. This synthetic phenotype suggests that Hbr1 plays a role in septum formation. In support of this, the abnormal filaments resembled cells depleted of Gin4 in the presence or absence of a *SWE1* deletion [36]. Interestingly, under embedded conditions, a *SWE1* null mutant forms only smooth colonies composed of chains of short pseudohyphal cells that have appropriately placed septa [42]. Therefore, the inability of the *HBR1/hbr1* strain to form proper septa is likely due to septin protein modifications or organization rather than a morphogenesis or cell size checkpoint.

In contrast, embedded *efg1/efg1* cultures showed three distinct phenotypes that were dependent upon cell depth, suggesting a role for O_2_ limitation. The deeper spindle colonies were inhibited from filament formation, consistent with a wild type farnesol response. Colonies in the upper half of the matrix contained pseudohyphal filaments producing opaque-like cells. However, in the mid-level colonies, a massive opaque cell synthesis occurred that spread by filament invasion into the surrounding agar. This phenotype has not been previously documented to our knowledge. Lindsay *et al* have shown that an *efg1/efg1 czf1/czf1* strain grown in liquid culture at 37°C switches to opaque cell morphology with some true opaque cell characteristics [9]. Farnesol addition to these cells resulted in significant cell killing, a phenotype shared by ‘true’ opaque cells [43]. However, growth under embedded conditions in the presence of farnesol did not reproduce the liquid assay in the development of opaque-type cells at 30°C [9]. These differences may reflect differences in incubation temperature, media composition, and assessment of filamentation.

The massive conversion of *EFG1* null cells to opaque-type cells may have implications for mating reactions within a host. In the proper environment, *in vivo* conversion of this magnitude may increase the probability of successful mating reactions due to sheer numbers. Indeed, mating can occur in the gastrointestinal tract as well as under anaerobic conditions [43]. However, we do not know whether these cells are mating competent or whether they only partially possess opaque cell characteristics as has been previously described [41]. Nonetheless, we demonstrate that *C*. *albicans* cells heterozygous for the mating-type locus can undergo an opaque cell conversion.

Hyphal growth during normoxia requires the down-regulation of Ace2-target genes required for cell separation by CDK phosphorylation of Thr^179^ on Efg1 and is consistent with filament separation defects in Egf1-depleted cells [44]. However, Ace2 but not Efg1 is required for hypha formation under oxygen-limited or embedded conditions. Mulhern *et al* propose that this is based on an increase in glycolytic and a decrease in respiratory genes, both of which are under Ace2 regulatory control. Under hypoxic or embedded growth conditions, decreased respiration was proposed to induce filamentation [45]. Indeed, Efg1 is a regulator of glycolytic and respiratory genes [11]. Thus, the RAM and PKA pathways regulate a common set of genes required for morphogenesis, indicating they are metabolically linked [46, 47]. The filamentation of *HBR1/hbr1* and *egf1/efg1* cells at all levels in the agar matrix and their lack of hyphae under aerobic conditions on Spider and GlcNAc agar are consistent with altered metabolic sensing. In support of this, hypoxia acted as a suppressor and restored invasive filament formation to *HBR1/hbr1* surface colonies. Together these data suggest that Hbr1plays a role in the balance between mitochondrial and non-mitochondrial metabolism that is regulated by Ace2.

Spindle-shaped colonies that form under embedded conditions have been reported previously [10, 12–14, 30, 48–50]. However, only emanating filaments have been examined and not the parent colony itself. Although spindle shaped colonies could originate as a consequence of the physically-restrained environment in the deeper levels of the agar column, a subset of these spindles had a defined, self-limiting structure formed by hyphal aggregation. Fungal sporocarps are composed of hyphal aggregates controlled by the positioning, growth rate and direction of individual hyphae within a glucan matrix [51]. The spindles shown in this work appear to be such a structure. These hyphal aggregates are reminiscent of the extended hyphal networks that constitute *C*. *albicans* biofilms [52, 53] and those from the body of an infected *Caenorhabditis elegans* nematode [54]. Using scanning electron microscopy, a similar extended hyphal morphology was recently documented with yeast cells cultured at 37°C in undiluted serum. Interestingly, an extracellular matrix could be seen developing from the true hyphae and apparently served as a cohesive element for the hyphal aggregates. Hyphal aggregates such as these were suggested to play a role in blood borne *C*. *albicans* dissemination [55]. The ability to form aggregates of true hyphae is a phenotype unique to the *HBR1/hbr1* strain. These structures are reminiscent of fungal balls (bezoars or mycetomas) that can result in renal or and lower urinary tract occlusions.

In conclusion, these data define Hbr1 as an important morphogenic regulator in *C*. *albicans* that regulates virulence in a mouse candidemia model, similar to *efg1* and *flo8* [5, 10]. Whether pathogenesis defects are similar between these strains remains for further experimentation. Hbr1 is a negative regulator of filamentation in an embedded matrix but a positive regulator under aerobic conditions. Mutagenesis of Hbr1 has revealed four morphogenic phenotypes that are genetically separable and each of these structures has a counterpart either in a *C*. *albicans* wild type or mutant strain. We also identified a novel spindle-shaped structure containing compacted, unbranched hyphae that forms under embedded conditions.

## Materials and Methods

*C*. *albicans* strains used in this work are listed in Table 2. Strains were maintained using standard conditions on YPD medium at 30°C [56], and all media contained 80 μg/ml uridine as a supplement [57]. YPS agar was used for embedded hyphal growth [10]. It contained 1% yeast extract, 1% peptone, 1% sucrose and 1.5% granular agar (BD Biosciences) adjusted to pH 7.6 with NaOH before autoclaving. The pH differences of agars from different sources made this adjustment necessary. Spider (mannitol), SLAD (low nitrogen), Lee’s and GlcNAc-containing agar plates were made as previously described [58–61]. Cells were embedded in YPS agar by placing ~ 5000 exponential phase cells (OD 600 nm ~0.8) in 200 μl normal saline at the bottom of a 50 ml sterile polypropylene tube, and adding 30 ml molten YPS agar. The mixture was dispensed into a 100×15 mm petri dish (BD Biosciences) and incubated at 24°C. Where indicated, farnesol (Sigma, St. Louis) in DMSO was added to 50 μM as an 8 μl droplet at the top of the tube and washed into the cells with molten agar.

**Table 2:** *C*. *albicans* strains used in this study.

Strain	Genotype	Reference
BWP17	*ura3Δ*::*λimm434/ura3Δ*::*λimm434 his1*::*hisG/his1*::*hisG arg4*::*hisG/arg4*::*hisG*	[57]
DAY185	*As BWP17 except pHIS1*::*his1*::*hisG arg4*::*URA3*::*arg4*::*hisG*	[64]
HLC52	*ura3*::*1 imm434/ura3*::*1 imm434 efg1*::*hisG/efg1*::*hisG-URA3-hisG*	[5]
JKC19	*ura3*::*1 imm434/ura3*::*1 imm434 cph1*::*hisG/cph1*::*hisG-URA3-hisG*	[5]
CAMPR8	as BWP17 except *HBR1/hbr1*::*ARG4*	[18]
R8 PRO*	As CAMPR8 except *RPS1*::*pCIP20-URA3-HIS1*	This study
R8 WT	as CAMPR8 except *HBR1/hbr1*::*arg4Δ*::*HBR1-SAT1-Nor* ^*res*^	This study
R819S	as CAMPR8 except *HBR1/hbr1*::*arg4Δ*::*HBR1-G19S- SAT1-Nor* ^*res*^	This study
R821S	as CAMPR8 except *HBR1/hbr1*::*arg4Δ*::*HBR1-G21S- SAT1-Nor* ^*res*^	This study
R866R	as CAMPR8 except *HBR1/hbr1*::*arg4Δ*::*HBR1-K66R- SAT1-Nor* ^*res*^	This study
R8CTH	as BWP17 except *HBR1/HBR1*::*TAP-HIS1*	This study
R8XS	as CAMP R8 except *RP10/rp10*::*pExpCAT4-9-URA3*	This study

*Prototrophic strain. The plasmid pCIP20 was obtained from A. Brown [65].

Mutations in the *HBR1* gene were introduced into *C*. *albicans* strains BWP17 and CAMP R8 *via* the SAT Flipper system using plasmid pSFS1A [62]. DNA regions selected for recombination substrates upstream and downstream of the HBR1 ORF were generated by PCR and cloned into TOPO vector pCR2.1 TA (Invitrogen, Carlsbad, CA). All amplified fragments were sequenced to verify their identity. The 3’ downstream region was amplified using primers PN 379 *SacII* and PN 526 *SacI* to enable cloning into pSFS1A at these same unique restriction enzyme sites and generated pSF1A-R13. Two substrates were amplified from the 5’ upstream end to include (i) a region for gene disruption (primers PN334 *XhoI* and PN 333 *KpnI* and (ii) a region to add back the entire HBR1 ORF including the 300bp terminator region (primers PN 333 *KpnI* and PN335 *XhoI*). These DNA fragments were cloned into the *KpnI* and *XhoI* sites of pSF1A-R13 and resulted in construction of a gene disruption vector pSF1-R1KO and a reconstitution vector pSF1-R1AB. Single amino acid substitutions in *HBR1* were generated using the Quick Change XL system (Stratagene, Agilent Technologies, Santa Clara, CA) using the *KpnI-XhoI* fragments containing the *HBR1* ORF and terminator in vector pCR2.1 as template. Mutations were introduced into the *HBR1* P-Loop region using primers PN224 (G19S), PN266 (G21S) and PN185 (K66R). These mutated and WT DNA fragments were released by *KpnI-XhoI* digestion and cloned into pSF2-R1KO at the unique *KpnI-XhoI* sites in the vector. Integration of this cassette into the *HBR1* locus was accomplished by *Nco1-Sac1* digestion and gel purification of the fragment. The *Nco1* site is present in the genomic DNA just 3’ to the *Kpn1* cloning site and was used to ensure that only genomic DNA sequences were integrated into the promoter to prevent any mutagenic effects.

An Hbr1 carboxyl-terminal TAP tag cassette was constructed using PCR primers PN 420/421 and plasmid pFA-TAP-URA (FJ160456) as template. The cassette was integrated into the genome of strain BWP17 as described below. Four URA+ colonies were analyzed for fusion protein production by Western blotting using anti-TAP polyclonal antisera (CAB1001; Thermo Scientific) (S1 Fig). PCR primer sequences are listed in Table 3.

**Table 3:** PCR primer sequences used in this study.

Name	Sequence (5’ to 3’)	Notes
PN333	TCGGGAAGTTTCAGGGTACCCATGG	KpnI Flipper
PN334	TCAGGCTCTAACTCGAGTCTAGC	XhoI Flipper
PN335	TGGTGACAACTCGAGAATGGTTTGACG	XhoI Add Back Flipper
PN526	TTTTGAGCTCATTACACATACAATATGC	SacI Flipper
PN527	GCTTTGAGGACCGCGGATCTCATTATTC	SacII Flipper
PN185*	GATGCCAGGTTAGATACTTCGATTGTAGACG	HBR1 K66R mutation
PN224*	CAGGTACACCTTCTTGTGGGAAATCATCTC	HBR1 G19S mutation
PN266*	CAGGTACACCTGGGTGTTCTAAATCATCTC	HBR1 G21S mutation
PN442	GCTATCACAAGGTCCCGGTG	Detect; 81 nt 5’ of PN333
PN372	TGTTTTGTGGTGCCGTGCAAG	Detect; *SAP2* promoter
PN357	TGTGCACCTATCCGACCAAGG	Detect; Ca*SAT1*
PN615	TGCTGTGCGTGCTTTTATTC	Detect; 558 nt 3’ of PN526

*Paired with reverse complement

Strain R8MET that over-expressed *HBR1* under the control of the MET3 promoter was constructed by the introduction of plasmid *pEXPCAT 4–9* [18] into the *RP10* locus of strain CAMPR8 via unique *StuI* sites in the *RP10* gene.

Cells were prepared for electroporation by an overnight growth in YPD at 30°C, diluted 1:100 into YPD, and cultured 4h at 30°C. Preparation for electroporation was carried out as described [62] using 1 μg gel –purified DNA in a BTX ECM830 (Harvard Apparatus) electroporator at 270V for 11 msec. Cells were plated on YPD agar containing 200 μg/ml Nourseothricin (Jena Biosciences, Jena, Germany) and incubated at 30°C. Single colonies appearing after two days were streaked on Nourseothricin plates to obtain single colony isolates. Gene substitutions in strain CAMPR8 were screened for an ARG^—^phenotype to identify heterozygous mutants.

Identification of insertion sites was made using PCR amplification with primers that spanned potential recombination junctions. Genomic DNA was isolated using a standard protocol and the upstream and downstream junctions were confirmed using primer pairs PN372/442 for 5’ end and PN357/615 for 3’ end fusion detection.

Photomicrographs of cells within agar were performed with an Olympus IX70 inverted microscope (Olympus USA Center Valley, PA) connected to an Insight QE Monochrome digital camera (Digital Instruments Inc., Sterling Heights, MI). Fluorescence microscopy used an Olympus BX51 microscope (Olympus USA Center Valley, PA) connected to a Nikon DMX1200F digital camera interpreted with ACT-1 image processing software (Nikon USA, Melville, NY). Calcofluor white (Sigma, St. Louis) staining of chitin followed the procedure of Pringle [63]. Cellular DNA was visualized with 4', 6-diamidino-2-phenylindole (DAPI) incorporated into fluorescence mounting media (Vector Laboratories, Burlingame, CA).

For challenge of mice, *C*. *albicans* cells were grown overnight in YPD medium at 30°C with aeration. Cells were harvested by centrifugation at 2500 RPM for 2 minutes in a bench-top clinical centrifuge at room temperature. Cells were washed twice in sterile non-pyrogenic normal saline, counted using a hemocytometer, and suspended to 5×10^6^ and 1×10^6^ cells/ml in sterile saline. Outbred 6- to 8-week-old Balb/c female mice (18–22 g) were obtained from Charles River Laboratories (Wilmington, MA, USA). Care and feeding conditions followed procedures of animal protocol LP-022 in compliance with the guidelines established by the Animal Care and Use Committees of the National Cancer Institute. Mice were inoculated intravenously with 0.1 ml *C*. *albicans* cells in the lateral caudal tail vein. Clinical signs of illness in each mouse were evaluated twice daily, and mice that displayed severe signs were euthanized immediately by CO_2_ inhalation.

## Supporting Information

S1 Fig Expression of an Hbr1 C-terminal TAP tag in *C*. *albicans*. Four URA+ isolates of *C*. *albicans* strain BWP17 transfected with an HBR1-TAP-URA3 cassette were tested for fusion protein expression using polyclonal antisera CAB1001 (Thermo Scientific). Lysates in lanes 1,2 and 4 were positive for the 48662 Dalton fusion protein that migrates slower than its calculated molecular weight as described previously [20].
(EPS)
